# Dual Pedicle Mastopexy Technique for Reorientation of Volume and Shape After Subglandular and Submuscular Breast Implant Removal

**Published:** 2013-09-16

**Authors:** Raffi Gurunluoglu, Edward Kubek, Jamie Arton

**Affiliations:** Plastic and Reconstructive Surgery, Denver Health Medical Center, University of Colorado Health Sciences Center, Denver

**Keywords:** breast implant, capsule formation, dermaglandular pedicle, implant removal

## Abstract

**Background:** The purpose of this article was to report our experience in achieving satisfactory breast shape and volume using dual pedicle mastopexy technique after subglandular and submuscular breast implant removal. **Methods:** Breast implant-related problems in 51 breasts included capsular contracture (Baker grade III-IV), 76%; implant rupture/bleed, 41%; breasts undergoing repeat surgery more than once, 22%. The size of the breast implants removed ranged from 240 to 525 cc (average size: 320 ± 65 cc) (saline-filled, 40%; silicone-filled, 60%; subglandular, 40%; submuscular, 60%). Capsulectomy, implant removal, and dual pedicle mastopexy were performed for reconfiguration of breast shape and reorientation of volume. **Results:** Mean follow-up was 14.5 months. On average, 65.0% of breast implant volume was achieved. There was 1-cup reduction in brassiere size in 21 patients, and the cup size remained the same in 5 patients. Postoperative pain scores were no pain or mild pain in 26 patients who initially in the preoperative evaluation reported having mild pain (11), moderate pain (12), and severe pain (3). Overall patient satisfaction scores were 3 (neutral) in 1 patient, 4 (satisfied) in 12 patients, and 5 (very satisfied) in 13 patients. **Conclusion:** The dual pedicle mastopexytechnique provided a reliable way of reorienting breast volume and configuring breast shape in patients who opted to have implants removed without replacement. The results demonstrated that a pleasing outcome could be obtained using the described technique with additional benefits of elimination of breast tenderness and discomfort secondary to implant removal and/or capsulectomy.

Several studies detected significant benefits of breast augmentation surgery supporting the hypothesis that cosmetic breast augmentation can have a significant and profound positive impact on a woman's satisfaction with her breasts and her psychosocial and sexual well-being.^1^

However, implant-associated problems are not uncommon. Although significant progress has been made in diminishing capsular contracture following breast augmentation in recent years, its occurrence and associated breast discomfort/tenderness and deformity continue to challenge plastic surgeons. The etiology of capsular contracture is multifactorial and includes, but is not limited to, anatomical plane of implantation, elastomer surface, implant content, surgical technique, myofibroblast activity, biofilm, low-grade infection, foreign body reaction, and blood products.^2^^-^4

Reported rates of capsular contracture vary widely. A recent study by Blount et al^5^ reported a capsular contracture rate of 0.4% to 3.9%. However, the longer the implants were in place, the greater the cumulative risk of developing contracture and other related problems, which would suggest a direct correlation between when the implant is placed and the time to develop contractures.^6^

It is generally accepted that the incidence of Baker Grade III or IV capsular contracture will increase over time and ranges from 10% to 15% after 10 years.^7^

Traditional treatment options have included capsulotomy, capsulectomy, implant exchange, pocket conversion, and mechanical manipulation.^8^^,^^9^ Recently, clinical evidence suggests beneficial role of acellular dermal matrix in treating patients with capsule contracture.^8^,^10^^-^12 A new option has been proposed in a recent article, which entails simultaneous implant exchange with fat in revision implant surgery.^13^

A patient with a diagnosis of symptomatic capsular contracture or of implant rupture may be reluctant to undergo an implant exchange procedure because of anxiety about implants. Therefore, removal of breast implants without replacement may be warranted to eliminate the risk of recurrent problems or secondary to strong desire by patients facing significant complications.

Flaps may be useful to restore breast volume and shape in these cases.^14^ But, this modality necessitates additional donor site surgery with potential risks and complications that most patients do not wish to undertake.^15^ Most patients, however, remain quite concerned about the aesthetic outcome of implant removal without replacement. A prospective outcome analysis conducted by Peters et al^16^ on 100 consecutive women who requested explantation of their silicone gel breast implants demonstrated that near 50% of the 57 patients who did not receive any implants felt disappointed/mutilated following surgery.

Reconfiguration of breast shape and reorientation of missing breast volume after implant removal without replacement present a formidable challenge for plastic surgeons. The concept of utilizing a buried dermaglandular pedicle for this purpose after implant removal is relatively new, although various dermaglandular pedicles have been previously described for enhancement of breast projection and upper pole fullness along with mastopexy.^17^^-^20 Hönig et al^20^ used inferior pedicle autoaugmentation mastopexy after breast implant removal for the first time according to the published literature. In this study, results that were evaluated by an independent evaluator were considered good to excellent in the majority of cases. However, patients’ interpretation of the outcomes was not considered. In addition, differentiation between subglandular and submuscular implants was not made, and technical details in each scenario were not discussed. Therefore, there is a need not only for further studies to help validate the efficacy of using inferior dermaglandular pedicle in patients undergoing implant removal but also for more detailed operative description of the technique in case of subglandular and submuscular implants.

The purpose of this article was to report our experience in achieving satisfactory breast shape and volume using dual pedicle mastopexy technique after subglandular and submuscular breast implant removal. We focused on the operative technique and patient-reported surgical outcomes, using this technique in 51 breasts of 26 patients.

## PATIENTS AND METHODS

Twenty-six patients with significant breast implant-related problems who did not want new implant were operated on by a single surgeon (R.G.) between 2007 and 2012. Breast implant-related problems in 51 breasts included capsular contracture (grades III-IV), 76%; implant rupture/bleed, 41%; and breasts undergoing repeat surgery more than once, 22%. Standardized Baker scale was used to evaluate capsular contracture, combining appearance, texture, and tenderness into a 4-grade severity score^21^^,^^22^ (Table 1). Radiologic assessment using ultrasonography or magnetic resonance imaging was done preoperatively for confirmation of clinical diagnosis of capsular contracture, hematoma, identification of implant location, and ruling out implant bleed/rupture, if necessary.^23^ In addition, pain scores of patients were retrieved from patient charts preoperatively to compare with postoperative scores.

Fifty-one breasts (50 bilateral and 1 unilateral) received capsulectomy, implant removal, components separation, and reorientation of breast volume and reconfiguration of breast shape. Data were collected on breast implant characteristics (size, saline vs silicone, subglandular vs submuscular) (Table 2).

## PREOPERATIVE MARKING

**Figure 1:**
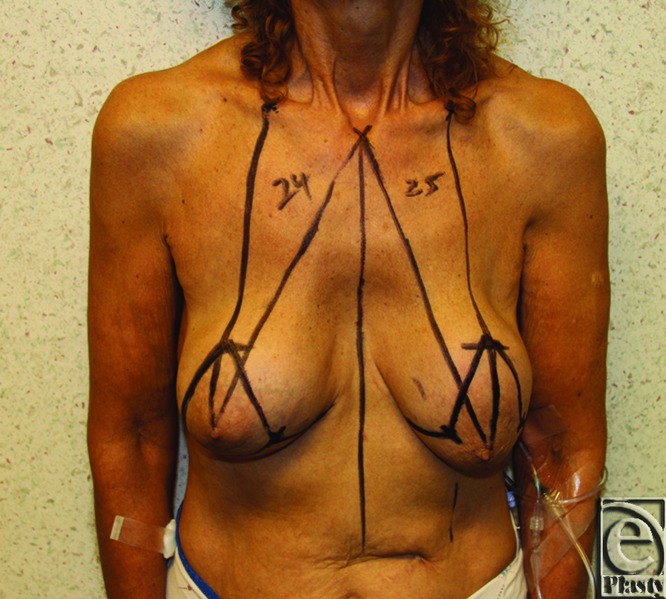
Preoperative marking photo in a 64-year-old patient showing wise pattern skin incisions. Top of the NAC was planned at 18 cm from the sternal notch. The final location of the nipple was determined intraoperatively after the reorientation of the breast volume using inferior dermaglandular flap and superomedial pedicle.

**Figure 2:**
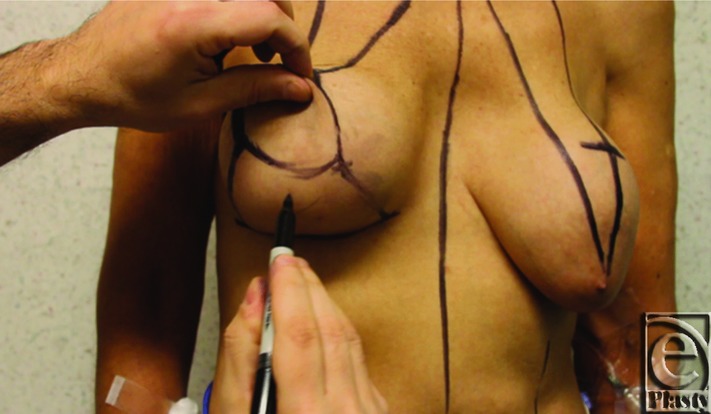
The width between the vertical skin incisions (and therefore the width of the dermal portion of the inferior dermaglandular pedicle) was determined by the amount of skin that could be excised to allow closure without tension both horizontally and vertically. Although this is checked preoperatively by pinch-test, it is safely confirmed and determined intraoperatively after breast implant removal. The height of the flap extends from the inframammary crease inferiorly to the superior medial pedicle superiorly. (Tip of the marker shows the inferior dermaglandular flap.)

Preoperative markings are performed with the patient in an upright position. The new nipple position is located on a point along the mid-breast line, approximately 18 to 21 cm from the sternal notch. In addition, the location for the top of the areola is usually marked at 1 to 2 cm above the inframammary crease. Markings are continued according to superior or superomedial pedicle technique and wise pattern skin incisions or vertical skin incisions with minor medial and transverse wedge extensions along the inframammary crease (Figs 1 and 2). The width between the vertical skin incisions (and therefore the width of the dermal portion of the inferior dermaglandular pedicle) is determined by the amount of skin that can be excised to allow closure without tension. Although this is checked preoperatively by pinch test, it is safely confirmed and determined intraoperatively after breast implant removal.

## OPERATIVE TECHNIQUE

### Subglandular implant

All patients receive perioperative antibiotics; most receive a first-generation cephalosporin. Usually, first the superior pedicle is de-epithelialized except for the nipple areolar complex (NAC). Then, a skin incision is made along the inferior edge of the superior pedicle regardless of location of breast implant, that is, subglandular versus submuscular implant. A dissection plane is developed first inferiorly staying close to the capsular tissue around the implant. The dissection is then continued circumferentially leaving the implant in place, if possible. This facilitates the dissection and removal of implant as well as capsular tissue en bloc. In addition, it reduces the risk of contamination of the operative site from the implant pocket. In case of subglandular implants, it is usually relatively easy to remove the posterior capsule. However, if the posterior capsule cannot be totally excised, scoring of the posterior capsule is performed to facilitate tissue adherence.

### Submuscular implant

In case of submuscular implants, it is important that the implant pocket is dissected without damaging the pectoralis major muscle. Therefore, after the skin incision is made along the inferior edge of the superior or superomedial pedicle, the dissection is first carried out inferiorly to identify the capsular tissue and implant pocket. Then the dissection is continued superiorly staying just above the capsular tissue. In this way, the inferior edge of the pectoralis major is easily identified. The muscle is then lifted up by means of retractors to further facilitate the dissection superiorly underneath the muscle. This maneuver prevents inadvertent injury to the pectoralis muscle. The dissection of the capsule is then completed in all directions leaving the implant in place, if possible. It may be difficult to remove the posterior capsule totally. In this case, scoring of the posterior capsule is performed to facilitate adherence of submuscular pocket. Closure of the submuscular pocket is reinforced using quilting sutures, preferably over a drain.

### Dual pedicle mastopexy technique

#### The inferior dermaglandular pedicle and medial and lateral breast pillars

After the implant is removed, the medial and lateral breast tissue and skin are evaluated to ensure tension free closure before making the medial and lateral skin incisions. The inferior pedicle is then de-epithelialized. The medial and lateral skin incisions are made toward the chest wall. In addition to its inferior base, it is essential to maximally keep soft tissue attachments of the dermaglandular flap to the chest to ensure maximal blood supply. Once these incisions are completed, medial and lateral breast pillars and the inferior dermaglandular pedicle are created. We roughly estimate the volume of the inferior pedicle by the following formula: volume = (width, cm) × (height, cm) × (length, cm).

**Figure 3:**
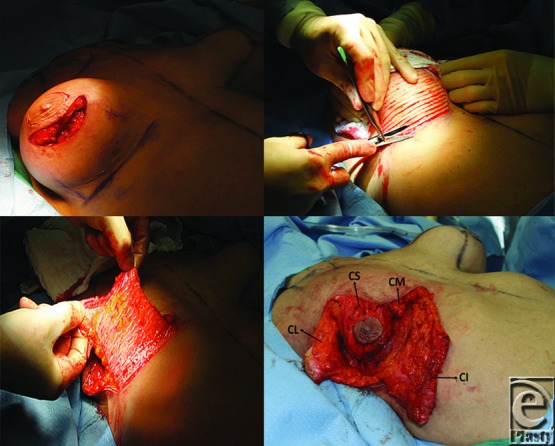
(Left above): A skin incision along the inferior aspect of the superomedial pedicle is made to perform capsulectomy and to remove breast implant. (Right above): Next, the inferior dermaglandular pedicle is de-epithelialized. (Left below): The inferior pedicle. (Right below): All components are separated after capsulectomy and subglandular implant removal (*CI: Component I*, inferior dermaglandular pedicle, *CM: Component M*, medial breast pillar, *CL: Component L*, lateral breast pillar, and *CS: Component S*, superomedial pedicle NAC).

**Figure 4:**
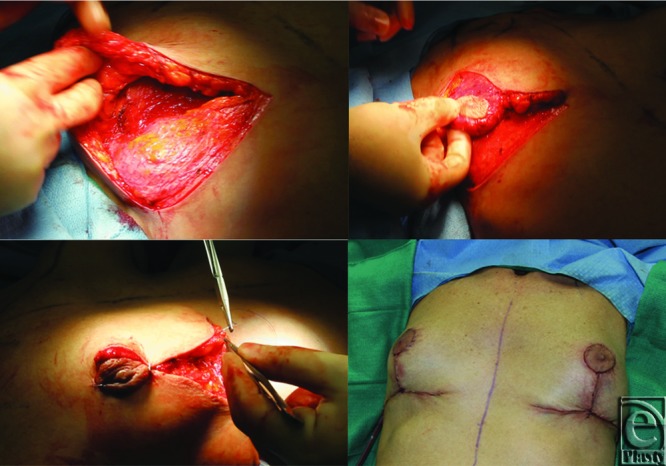
(Left above): Intraoperative picture: The inferior pedicle is simply placed in the subglandular pocket over the pectoralis major muscle and anchored to the pectoralis muscle fascia using a few absorbable monofilament type stitches under the breast parenchyma toward the upper pole. (Right above): Superior medial pedicle is placed on top of the dermaglandular flap. (Left below): Closure of the medial and lateral pillars over the flap assists to optimize upper pole fullness and narrows the breast base. (Right below): The final location of the NAC is determined with the patient sitting up at 90° on the operating table.

The dissection of the superior pedicle NAC is the last step. The superior medial pedicle is selected when nipple-areolar elevation is restricted and superior pedicle is not suitable because of firm, dense breast parenchyma, or the NAC needs to be moved a greater distance upward than the superior pedicle permits (Figs 3 and 4). In case of previous submuscular implants, an adequate pocket should be dissected under the superior pedicle and over the pectoralis muscle to accommodate the inferior pedicle.

#### Reorientation of breast volume and reconfiguration of breast shape

**Figure 5:**
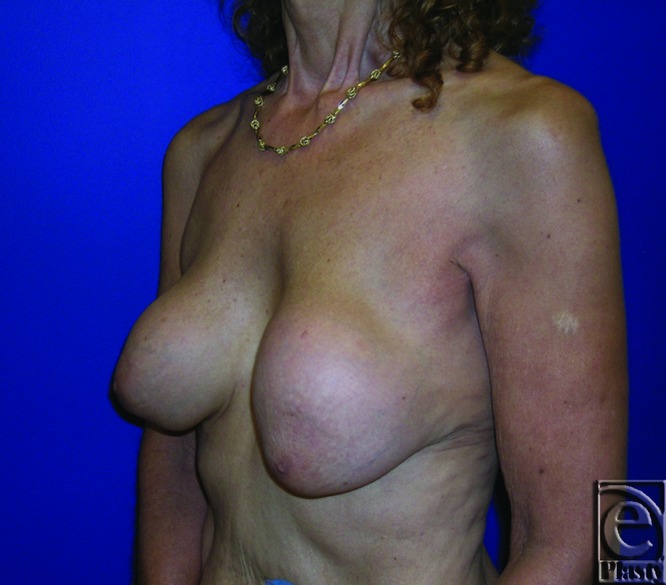
Preoperative view of the patient shown in the preoperative marking.

**Figure 6:**
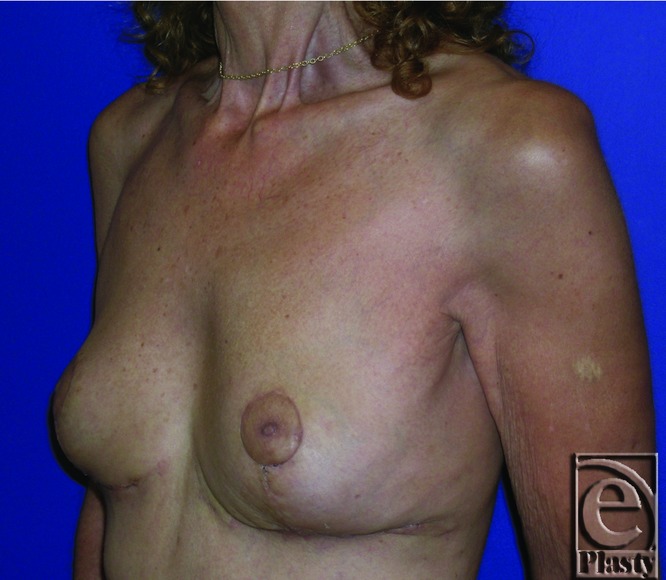
Early postoperative view of the patient discussed in the operative technique.

The existing breast tissue is reconfigured to produce the best possible shape with the superior or superior medial pedicle and the NAC on top and the de-epithelialized breast tissue underneath. In the case of previous subglandular implant pocket, the dermaglandular flap is simply placed in the subglandular pocket over the pectoralis major muscle, which results from the removal of breast implant. The flap is anchored to the pectoralis muscle fascia using a few absorbable monofilament type stitches under the breast parenchyma toward the upper pole. In the case of previous submuscular implant, the inferior pedicle is placed over the inferior portion of the pectoralis muscle in a subglandular location, dissected earlier. However, the size of the subglandular pocket should be adequate to accommodate the inferior pedicle. Closure of the medial and lateral pillars over the flap (Component I) assists to optimize upper pole fullness and narrows the breast base. Suction drains (15 French round Blake drain; Ethicon, Inc, Somerville, New Jersey) are placed in the standard fashion. Finally, the vertical and horizontal incisions are closed in layers. The final location of the NAC is determined with the patient sitting up at 90° on the operating table. A circular skin aperture is created after marking with a cookie cutter at the superior aspect of the vertical incision at a distance 5 to 6 cm from the inframammary crease and NAC is inset (Fig 4, right below). Figures 5 and 6 belong to the patient included in the preoperative planning section, which present preoperative and early postoperative appearance of the patient, respectively (See the Supplementary video for operative technique in a case after subglandular breast implant removal, available at:

).

## PATIENT 1 (A CASE OF SUBGLANDULAR IMPLANT)

**Figure 7:**
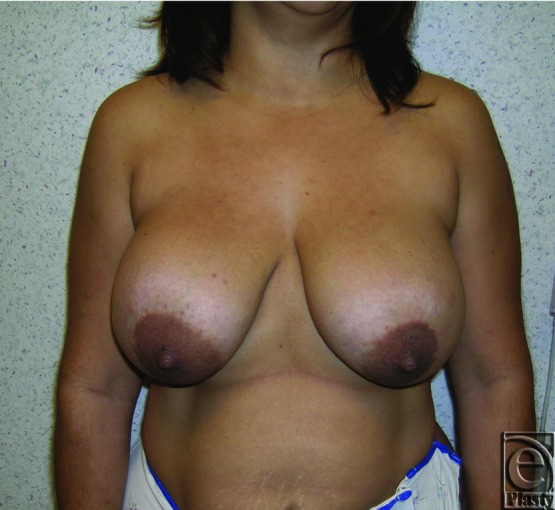
Preoperative frontal view of patient 1 (Baker grade III) with subglandular implant.

**Figure 8:**
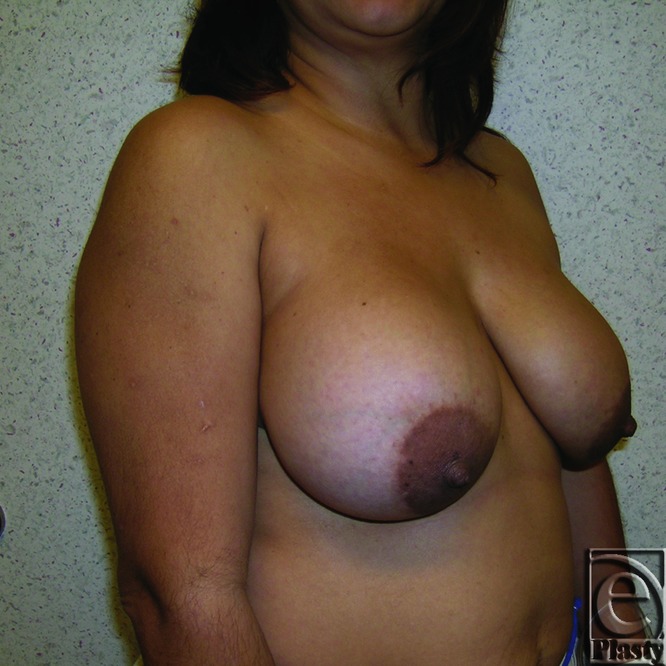
Preoperative right oblique view of patient 1 (Baker grade III) with subglandular implant.

**Figure 9:**
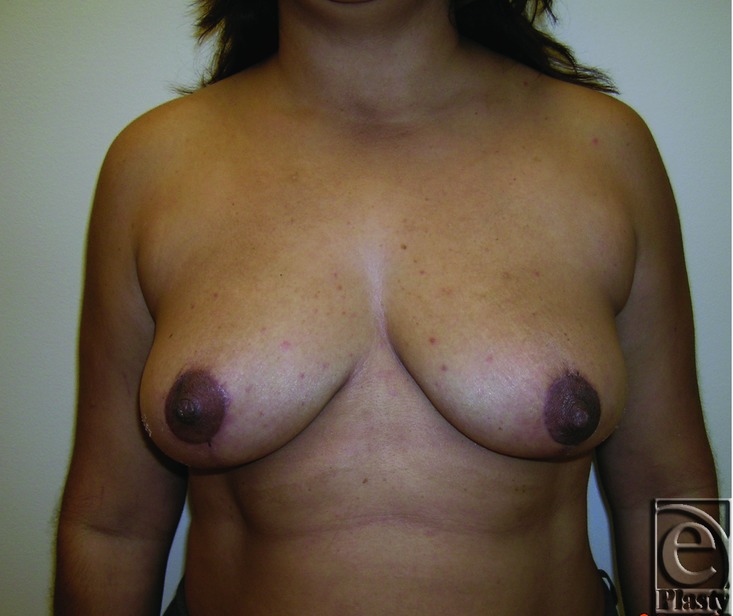
Postoperative frontal view of patient 1 at 12 months after undergoing capsulectomy, breast implant removal, and dual pedicle mastopexy technique.

**Figure 10:**
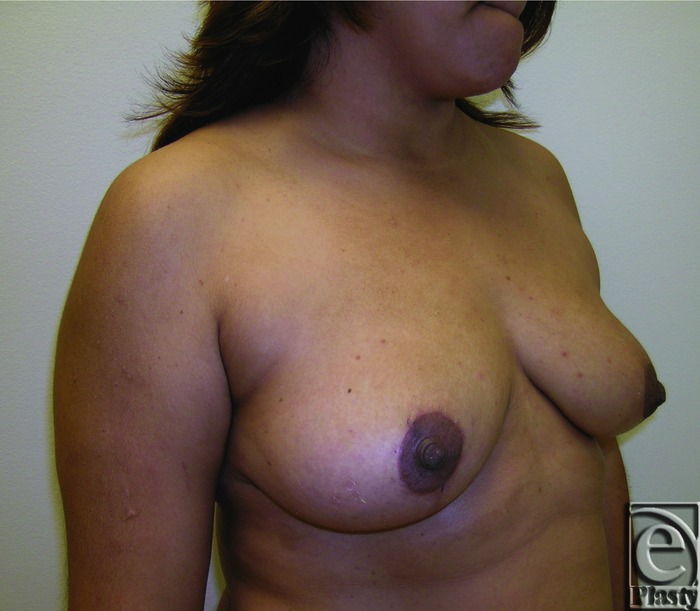
Postoperative right oblique view of patient 1 at 12 months.

A 38-year-old patient with a history of breast augmentation presented with moderate right-breast tenderness present for the past 8 months. She had 525-cc-saline implants in subglandular location. She was classified as Baker III (Figs 7 and 8). Sternal notch to nipple distance was 28 cm on each breast. Preoperative brassiere size was 36 cc. Standard surgical options, including capsule resection and implant exchange with or without pocket conversion, were discussed in detail on 2 separate occasions prior to final surgical decision. She strongly desired her implants to be removed. She opted to undergo bilateral implant removal, capsulectomy, and reorientation of breast shape and volume, using components separation procedure following extensive preoperative counseling. Postoperative follow-up at 12 months showed aesthetically pleasing outcome. Her brassiere size was 36B and sternal notch to nipple distance was 20 cm (Figs 9 and 10).

## PATIENT 2 (A CASE OF SUBMUSCULAR IMPLANT)

**Figure 11:**
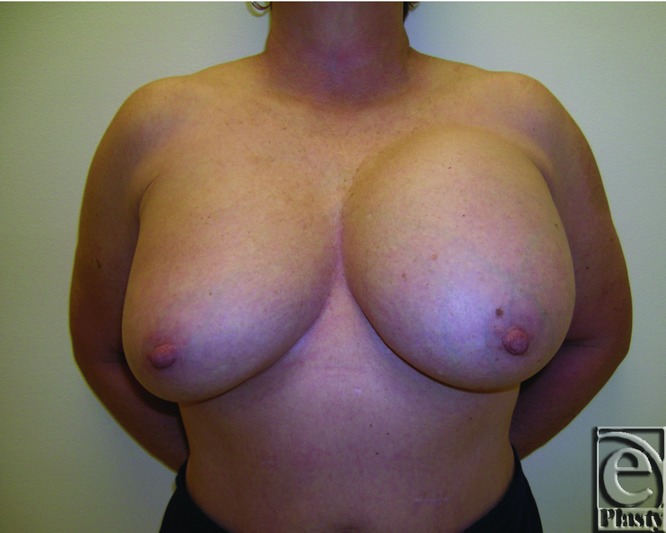
Preoperative frontal view of patient 2 (left breast Baker grade IV) with submuscular implant.

**Figure 12:**
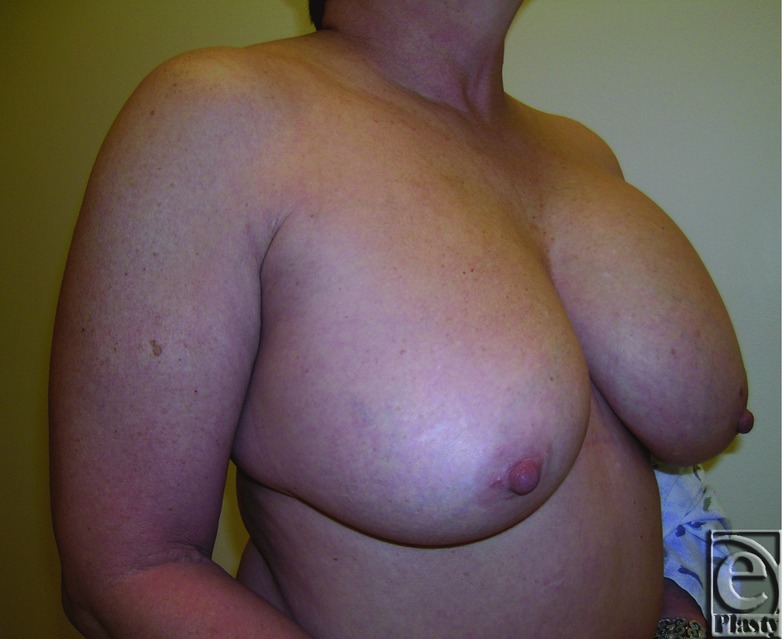
Preoperative right oblique view of patient 2 (left breast, Baker grade IV) with submuscular implant.

**Figure 13:**
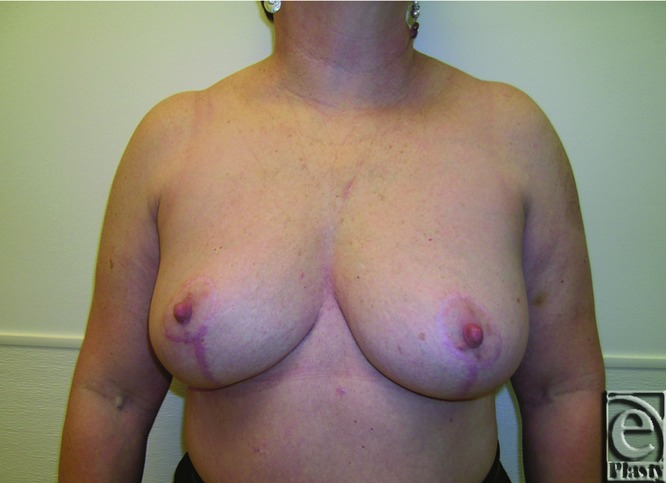
Postoperative frontal view of patient 2 at 14 months after undergoing capsulectomy, breast implant removal, and dual pedicle mastopexy technique.

**Figure 14:**
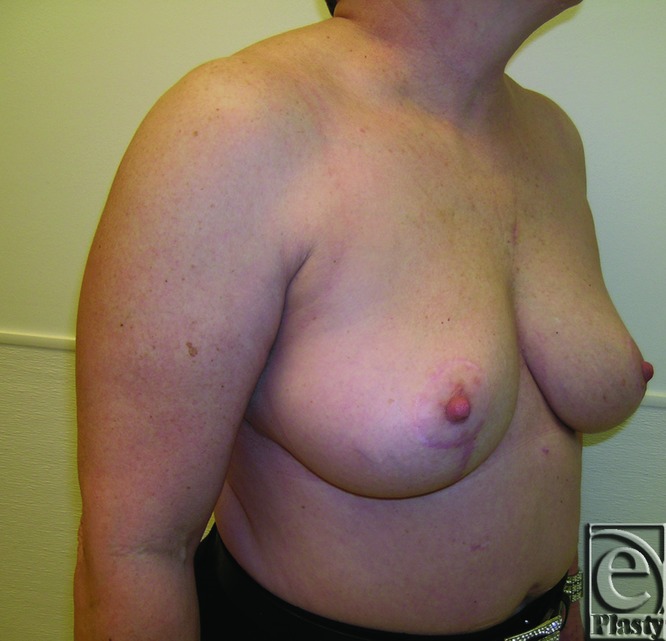
Postoperative right oblique view of patient 2 at 14 months.

This was a 57-year-old patient who presented with a left breast deformity and severe tenderness present for 3 months. She had undergone bilateral breast augmentation 30 years ago. Imaging studies showed hematoma formation in addition to thick capsule formation around the implant on the left breast. She was classified as grade IV according to Baker classification (Figs 11 and 12). Her preoperative brassiere size was 38D. She strongly wanted to have her implants removed and rejected any other major reconstructive surgery. She received bilateral implant removal, capsulectomy, and components separation procedure for reorientation of breast shape and volume. Saline-filled breast implants, 270 cc, were removed from subpectoral sites. Her postoperative brassiere size remained as 38D. Postoperatively, sternal notch to nipple distance was 21 cm as opposed to 28 cm, preoperatively (Figs 13 and 14).

## RESULTS

Patients’ age ranged between 38 and 57 years with a mean of 48 years. The average time from the last breast augmentation surgery to breast implant removal and components separation procedure was 20 years 6 months (range: 10–33 years). Eleven patients had a history of revision breast surgery with reimplantation between 10 and 16 years ago. The size of the breast implants removed was between 240 and 525 cc (average size: 320 ± 65). Forty percent of the implants were saline, and 60% were silicone-filled. Forty percent of the implants were removed from the subglandular plane, whereas 60% were in the submuscular location. All patients but one (periareolar incision) had previous inframammary incision scars ranging from 3 cm to 5 cm. The inferior pedicle was safely utilized in patients with previous inframammary incision scars. Similarly, previous periareolar incision scar did not preclude utilization of this technique. Reorientation of breast volume using the inferior pedicle ranged between 120 and 320 cm^3^ (average volume: 210.0 ± 50). We roughly estimated the volume of the inferior pedicle by the following formula: volume = width (cm) × height (cm) × length (cm). By using the formula: (average volume of the inferior dermaglandular pedicle = 210 cm^3^)/(average volume of the breast implant = 320 cm^3^ × 100 = 65%), we calculated that on average, 65.0% of breast implant volume was replaced. There was 1-cup reduction in brassiere size in 21 patients, and the cup size remained the same in 5 patients. Sternal notch to nipple distance ranged between 19 and 22 cm with an average decrease of 6.5 cm. There were no complications related to the NAC. Two patients underwent exploration and hematoma evacuation of the left breast in the early postoperative period. One patient suffered from a superficial wound at the T-junction, which healed with local wound care in 4 weeks. In another patient, hypertrophic scars have formed along the incisions, which were treated with silastic sheet with good response in the long term. Mean follow-up was 14.5 months. Postoperative pain scores were no pain or mild pain in 26 patients who initially in the preoperative evaluation reported having mild pain (11), moderate pain (12), and severe pain (3). Overall patient satisfaction score were 3 (neutral) in 1 patient, 4 (satisfied) in 12 patients, and 5 (very satisfied) in 13 patients.

## DISCUSSION

We would like to begin our discussion emphasizing the fact that the described technique may be considered in patients with significant implant-associated problems who no longer want new breast implant and/or any major reconstructive surgery.

The dual mastopexy technique utilizes in situ dermaglandular flap as a practical option by taking volume that was already there for reorientation of breast volume after implant removal. In addition, medial and lateral breast pillars were used along with superiorly based NAC to obtain a breast shape with narrower base and more appropriately positioned NAC. In addition, the optimal approximation of medial and lateral pillars helps reduce the potential dead space particularly in the case of relatively larger-size pocket and smaller inferior pedicle.

From technical standpoint, it is critically important to differentiate between subglandular and submuscular implants for an optimal outcome when utilizing this technique. This requires both preoperative assessment and intraoperative awareness and vigilance. During dissection of the implant pocket superiorly, staying close to the overlying capsule allows differentiation between submuscular and subglandular pocket and avoids inadvertent injury to the pectoralis major muscle.

Another critical technical point to consider is the location of the skin incisions medially and laterally over the breast. While preoperative markings provide intraoperative guidance, where to place the incisions precisely is determined after implant removal and ensuring that the skin can be closed without undue tension over the buried dermagladular flap.

A significant observation in this series is that a preexisting submammary scar previously used for breast augmentation did not preclude the utilization of the inferior pedicle. All the inferior dermaglandular flaps remained viable, which was confirmed by intraoperative assessment as well as by the absence of clinical and radiologic evidence of fat necrosis at long-term follow-up evaluation. This may be attributed to relatively short preexisting scars, to sufficient time that has elapsed since the time of original augmentation procedure, and to increasing the horizontal width of the pedicle beyond the scar tissue both medially and laterally. Average time between breast augmentation and implant removal, along with the described surgery, was 20 years and 6 months in this series. Most patients had their implants placed during early adulthood period. And, it is likely that most patients in our series experienced breast enlargement and ptosis over time following pregnancies and weight gain during middle age. These possible changes occurring over time in our patients who had undergone breast augmentation many years ago enabled us to take the advantage of the enlarged soft tissue envelope to reshape the breast and restore the volume even after removal of sizable breast implants. However, it should be noted that there was no net increase in breast volume in any of our patients. By transposing existing breast tissue in the lower pole, some portion of the volume loss secondary to implant removal was replaced. With this procedure, breast tissue was taken from the area where there was excess to the area where there was a deficit after implant removal. In this way, on average 65.0% of breast implant volume was replaced. In the majority of patients (81%), there was 1-cup reduction in brassiere size as this would be expected. However, 19% of patients claimed that their brassiere size did not change significantly after the surgery. This may be related to removal of relatively smaller implants in full-framed large-breasted patients in whom net decrease in size was not significant enough to change their brassiere size. Other plausible explanations for the brassiere size remaining the same in 5 patients may be due to patients’ inaccurate subjective assessment of their own brassiere size or switch to use a different brassiere brand having cup sizes different from what they used to wear.

Full-framed, full-breasted patients (such as patient 2) with a modest degree of preexisting breast ptosis who had undergone only modest augmentations and who might have gained weight since surgery had good aesthetic outcomes following implant removal and configuration of breast shape and reorientation of breast volume, using the dual pedicle mastopexy technique. We also obtained pleasing results in patients with a substantial amount of breast tissue and relatively large-volume augmentations (patient 1). It is our impression that pleasing results may also be obtained in patients of asthenic body configuration and older patients with little amount of breast tissue over relatively large implants (patient presented in the operative technique section). Moderate degree of ptosis in most of these patients allows utilization of adequate amount of dermaglandular pedicle for reorientation of breast volume. The problem, however, may arise in some of these individuals when they have a relatively short distance between the areola and inframammary fold, jeopardizing the utilization of optimal amount of tissue. Therefore, superior pole fullness that could be offered with breast implant will not be maximized in these patients. These patients will not be good candidates for the described technique.

The majority of patients undergoing surgery were satisfied with the outcome with a particular emphasis on breast using a 5-point satisfaction scale. We cannot be sure if high level of patient satisfaction could simply be attributed to successful treatment of the complications or be singularly attributed to the use of the described technique. However, overall patient satisfaction scores with particular emphasis on breast morphology and size have indicated that the favorable outcomes in breast shape and volume can be obtained in women who refused to have new implants.

In addition, we also demonstrated improvement of breast tenderness/discomfort by making comparative analysis between preoperative and postoperative pain assessment. We demonstrated that for women with significant implant-related problems who did not want another implant, removal of the breast implants did not appear to result in disappointing and unsatisfactory outcome. In fact, patient satisfaction scores and elimination of breast tenderness/discomfort in most patients undergoing this procedure have shown that in practice the described technique may provide a reasonably good alternative treatment after implant removal without replacement.

## CONCLUSIONS

The dual pedicle mastopexy provided a reliable way of reorienting breast volume and configuring breast shape following removal of breast implants without replacement. The favorable outcomes support the potential use of the described technique in patients with similar problems. We feel that the results of our study allow us to offer patients an optimistic view of postoperative results. However, further studies may be helpful to support the findings of this study.

## Figures and Tables

**Table 1:** Implant-related complications in 51 breasts of 26 patients

Breast	Capsular contracture	Implant rupture/bleed	Repeat surgery
*Baker scale*			
N = 51	III (n = 18)	n = 21	n = 11
	IV (n = 21)		
Percentage	III (35.2%)	(41.1%)	(21.5%)
	IV (41.1%)		

**Table 2:** Characteristics of breast implants

Features	Breast implant	
Location	Subglandular, 40%	Submuscular, 60%
Type	Saline, 40%	Silicone, 60%
